# Foamy virus zoonotic infections

**DOI:** 10.1186/s12977-017-0379-9

**Published:** 2017-12-02

**Authors:** Delia M. Pinto-Santini, Carolyn R. Stenbak, Maxine L. Linial

**Affiliations:** 1 Division of Basic Sciences, Fred Hutchinson Cancer Research Center, Seattle, WA USA; 2 Biology Department, Seattle University, Seattle, WA USA; 3 Division of Basic Sciences, Fred Hutchinson Cancer Research Center, 1100 Fairview Ave. N., A3-205, Seattle, WA 98109 USA

**Keywords:** Foamy virus, Simian foamy virus, Retrovirus, Nonhuman primates, Zoonoses

## Abstract

**Background:**

Foamy viruses (FV) are ancient complex retroviruses that differ from orthoretroviruses such as human immunodeficiency virus (HIV) and murine leukemia virus (MLV) and comprise a distinct subfamily of retroviruses, the Spumaretrovirinae. FV are ubiquitous in their natural hosts, which include cows, cats, and nonhuman primates (NHP). FV are transmitted mainly through saliva and appear nonpathogenic by themselves, but they may increase morbidity of other pathogens in coinfections.

**Conclusions:**

This review summarizes and discusses what is known about FV infection of natural hosts. It also emphasizes what is known about FV zoonotic infections A large number of studies have revealed that the FV of NHP, simian foamy viruses (SFV), are transmitted to humans who interact with infected NHP. SFV from a variety of NHP establish persistent infection in humans, while bovine foamy virus and feline foamy virus rarely or never do. The possibility of FV recombination and mutation leading to pathogenesis is considered. Since humans can be infected by SFV, a seemingly nonpathogenic virus, there is interest in using SFV vectors for human gene therapy. In this regard, detailed understanding of zoonotic SFV infection is highly relevant.

## Background

Many human pandemics, including those caused by HIV-1, a retrovirus, and influenza A, an orthomyxovirus, originated from zoonotic infections. It is thought that simian foamy viruses (SFV) are more frequently transmitted from nonhuman primate (NHP) hosts to humans than are other retroviruses, and as a result, SFV zoonotic transmissions have been monitored for several decades (previously reviewed in [1–3]). We provide an updated overview of foamy virus (FV) zoonotic transmission and its implications for human health.

Based on viral molecular properties, retroviruses (Retroviridae) have been subdivided into two subfamilies, the Orthoretrovirinae, including alphaviruses, gammaviruses, and lentiviruses, and the Spumaretrovirinae, including foamy viruses [4]. FV apparently existed before their closest relatives Orthoretrovirinae and Hepadnaviridae (hepatitis B viruses) [5]. Spumaretrovirinae are endemic in many mammalian hosts including cats, cows, horses, bats and NHP, but not in humans. The prototype foamy virus (PFV) was originally thought to be a human virus since it was isolated from a human nasopharyngeal cancer cell line [6]. Once the PFV genome was sequenced, and compared to the sequence of a chimpanzee SFV it became clear that PFV was of chimpanzee origin [7]. All current evidence indicates that PFV is the result of a chimpanzee FV zoonotic infection in the Kenyan from whom the nasopharyngeal cancer cell line was derived.

All NHP species examined to date, including New World monkeys (NWM), Old World monkeys (OWM) and apes, are infected by SFV [8]. Thus far, there is no observed pathogenicity associated with SFV infection in any natural host. FV transmission occurs mainly through saliva and all natural hosts are known to share saliva via biting, grooming and/or food sharing. Other natural transmission routes, if they exist, have not been identified. However, it has been shown that blood transfusion from an infected to an uninfected nonhuman primate does lead to infection [9]. Thus, in natural or research settings, exposure of uninfected animals to a large amount of infected blood could lead to infection. As seen in natural hosts, humans zoonotically infected with SFV show no signs of associated disease.

## Foamy virus genome structure and replication

FV are complex retroviruses that share *gag*, *pol* and *env* genes with orthoretroviruses. However, there are many distinct features of FV that are reminiscent of hepatitis B virus (HBV) and other Hepadnaviridae. For example, the FV Pol protein (polymerase or reverse transcriptase) is translated from its own AUG, rather than as part of a Gag-Pol fusion protein as is the case for orthoretroviruses [10]. Secondly, completion of reverse transcription occurs within the virion, prior to infection of a new host cell, making the functional FV genome double-stranded DNA rather than single-stranded RNA [11]. Because of these features, which are unique among retroviruses, FV have been classified as a separate subfamily of Retroviridae.

**Fig. 1:**
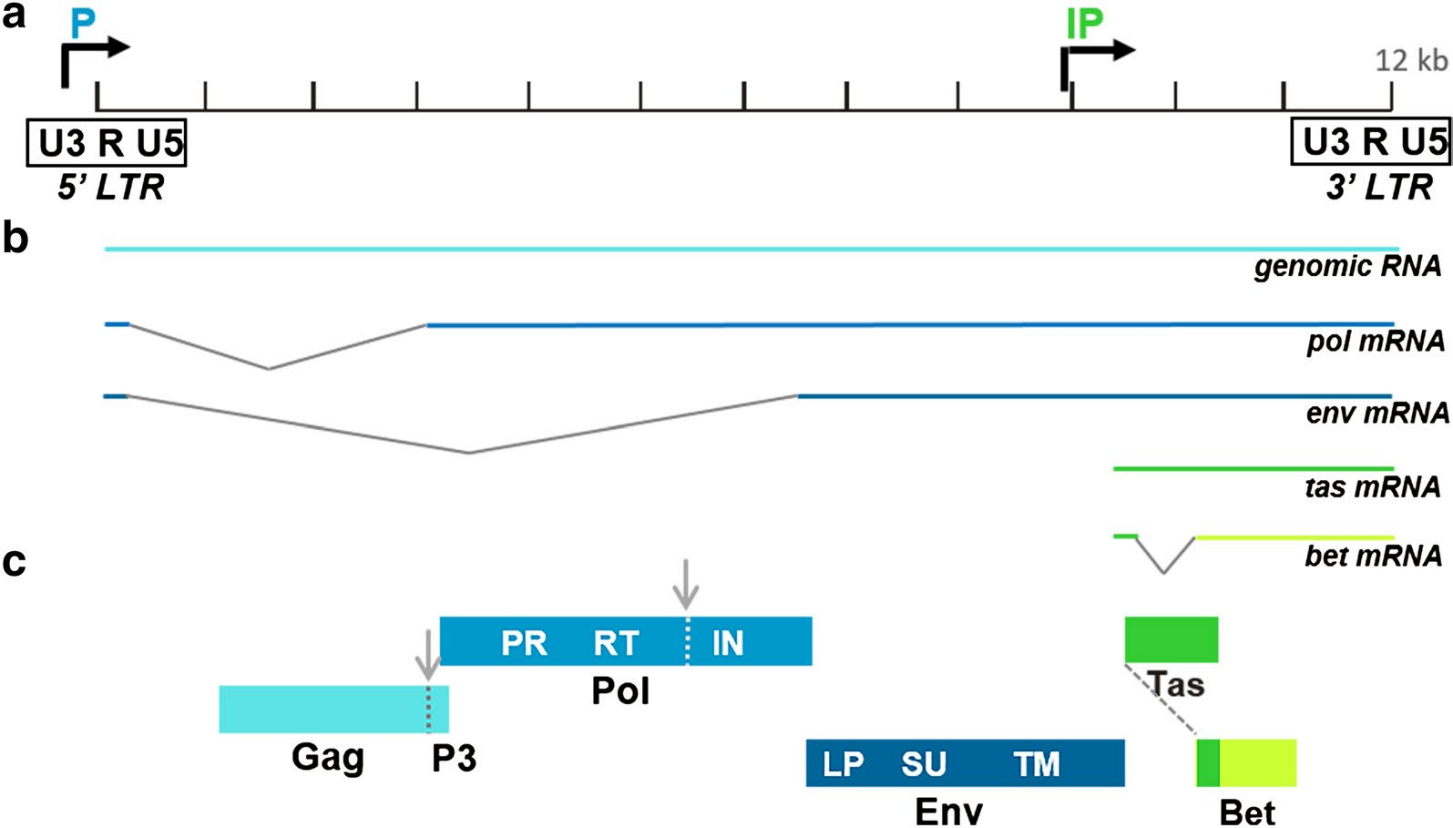
The Prototype Foamy Virus (PFV) genome, RNA transcripts, and protein products. **a** The molecular clone PFV-13 is depicted (Genbank accession no. U21247; 11,954 bp). The proviral long terminal repeats (LTR) are indicated at the 5′ and 3′ ends of the genome. Each LTR is composed of U3, R and U5 sequences. The U3 sequences are from the 3′ end of the viral RNA genome and the U5 sequences are from the 5′ end of the viral RNA genome. The R sequences are repeat sequences that are created during reverse transcription. Horizontal arrows indicate the location of the two viral promoters. The 5′ LTR promoter is blue and indicated as “P” while the internal promoter is green and indicated as “IP”. **b** The five major PFV mRNAs are shown. The first three mRNAs, including the unspliced genomic RNA and the spliced *pol* (polymerase) and *env* (envelope glycoprotein) mRNAs, are expressed from the 5′ LTR promoter and colored different shades of blue. The full-length unspliced RNA (light blue) serves as both the viral genome and the mRNA for the Gag (viral capsid) protein. The two smaller PFV mRNAs encoding the accessory proteins Tas (transactivator) and Bet proteins originate from the IP and are colored dark and light green, respectively. **c** The shaded boxes indicate the major PFV protein products, Gag, Pol and Env, as well as Tas and Bet. Viral protease-mediated cleavage sites within Gag and Pol are indicated with dashed lines and vertical arrows. The C-terminal P3 domain, released upon Gag cleavage, is indicated. The Pol protein contains PR, the protease domain, RT, the reverse transcriptase domain, and IN, the integrase domain. The Env protein is comprised of LP, leader peptide domain, SU, surface domain and TM, transmembrane domain

The prototype foamy virus (PFV) genome is shown in Fig. 1a. The *gag*, *pol* and *env* genes are arranged in that order from the 5′ end of the genome. The long terminal repeat at the 5′ end of the provirus (5′LTR) contains the viral promoter and enhancers that drive transcription of the *gag*, *pol* and *env* mRNAs. The *pol* and *env* mRNAs originate from the 5′LTR and are spliced (Fig. 1b). In addition, PFV contains an internal promoter (IP) that controls transcription of RNAs encoding the accessory proteins Tas and Bet. PFV mRNAs and proteins are shown in Fig. 1b, c, respectively. Tas is the transcriptional activator required for transcription from the 5′LTR. Tas also up-regulates transcription from the IP, but a basal level of transcription of *tas* and *bet* mRNAs from the IP occurs in the absence of Tas. As the Tas protein accumulates, the 5′LTR is activated [12]. Although the second non-structural protein, Bet, is highly expressed, its function is still not well understood [13–19]. Naturally-infected NHP produce antibodies that react strongly with both Gag and Bet proteins when assayed by Western blot, and the presence of anti-Gag and anti-Bet antibodies has proved useful for the detection of FV infections in vivo.

In HIV and other orthoretroviruses, the Gag precursor protein is cleaved into at least 3 Gag proteins, Matrix (MA), Capsid (CA) and Nucleocapsid (NC). The MA protein is myristylated and is required for interaction with the Envelope glycoproteins (Env) and for infectivity. The CA protein is the major virion structural protein, while the NC protein interacts with the RNA genome and is involved in its encapsidation. In contrast to HIV, FV Gag is not cleaved into MA, CA and NC, yet it does contain domains with functional similarity to each of the three mature HIV Gag proteins. Although they serve the same function, the NC domains of orthoretroviruses and foamy viruses are very different at the sequence level. Overall, the amino (N) terminus of the FV Gag protein is more similar to the Gag proteins of orthoretroviruses than is the C terminus, reviewed in [20]. The only cleavage of FV Gag occurs close to the C terminus, releasing a ca. 3 kDa peptide, P3 (Fig. 1c). When a mutation was made that removed the FV Gag cleavage site so that only the full-length Gag protein was produced, the virus was not infectious [21]. It is possible that cleavage is required to change the conformation of the cleaved Gag protein and thus its function, but this has not been demonstrated. It is not known whether the P3 peptide itself plays any role in replication. Since only about half of the synthesized Gag proteins are ultimately cleaved, this results in the presence of a Gag doublet in Western blots.

Retroviruses are known to have a high mutation rate. However, FV are unusual in that their genomes are highly conserved between individuals of the same species and over time, compared to those of orthoretroviruses [22]. Mutations in retroviral genomes are largely attributed to an error-prone reverse transcriptase (RT). The fidelity of PFV RT has been examined in vitro and in cell culture. Recombinant PFV RT was found to have a nucleotide substitution rate very similar to that of recombinant HIV-1 RT in vitro [23]. However, point mutations during replication were less frequent in PFV compared to HIV-1 in cell culture, suggesting a higher fidelity for PFV RT than for HIV-1 RT in vivo [18]. The basis for the lower in vivo mutation rate of PFV RT is unknown.

While a higher fidelity RT would support the genome stability observed for FV, Gartner et al. [18] also found that PFV recombination through template switching was a frequent event. This is important because, like an error-prone RT, recombination can also contribute to virus evolution. Sequence analyses of the *gag* and *env* genes in SFV-infected Old World monkeys (OWM) has identified recombinant viruses, supporting the idea that recombination also occurs in natural infections [24, 25]. Template switching and recombination during reverse transcription, along with the documented cross-species transmission of FV in NHP, leads to the concern that viral recombination between FV of different host species could occur in a co-infected animal [26]. There is evidence for co-infection with more than one SFV species [27, 28], but no evidence to date of humans infected with more than one SFV species, nor for humans infected with a recombinant SFV derived from more than one species. However, it is possible that FV recombination events could occur similar to those that led to the emergence of the retroviral human pathogen HIV [29].

Cells productively infected by all retroviruses, including foamy viruses, synthesize a large amount of viral mRNAs that encode the viral proteins. These mRNAs include genomic length mRNAs that encode the Gag protein. For each packaged genomic RNA, there are thousands of *gag* mRNAs. Thus, RT-PCR from infected cells detects primarily mRNA, which is indicative of active viral replication. FV, like all orthoretroviruses, package genomic RNA into viral particles. However, in the case of FV, reverse transcription occurs within viral particles as they bud from cells [11] leading to DNA genomes in the particles.

PFV replicates in many primary cells and established cell lines, irrespective of the species from which the cells were isolated. In tissue culture cells in which viral replication is robust, the virus often induces cytopathic effects (CPE). However, human hematopoietic cell lines can be infected by PFV after co-culturing with infected adherent cells, and in these cells the virus can replicate to high titers without CPE [30]. In naturally infected hosts, such as macaques or *Cercopithecus*, SFV replication is seen only in specialized cell types such as superficial differentiating epithelial cells of the oral mucosa [31, 32]. Infected NHP typically have low levels of latent proviral DNA in most tissues, while SFV RNA is detected only in the oral mucosa. Buccal swabs from natural hosts can therefore be used to isolate viral RNA and obtain sequences of actively replicating FV. PCR detection of FV DNA in PBMC is often used to identify infected individuals and determine proviral DNA sequences. FV establishes lifelong, persistent infection and can be transmitted efficiently within natural host populations primarily through saliva, which contains infectious viruses (reviewed in [4]). Viral RNA is never seen in PBMC freshly obtained from infected animals [31, 32] but latently-infected PBMC obtained from FV positive animals will produce virus when stimulated to divide in tissue culture [33].

Given the lack of pathogenicity in natural and human hosts, as well as stable integration of the viral genome into host chromosomes, FV are of interest as vectors for gene therapy applications (reviewed in [34]). FV have additional advantageous features for their use as vectors, including a functional DNA genome which might increase virion stability and a large genome size which allows for insertion of up to 9 Kb of DNA. FV vectors have successfully been used to treat genetic diseases in dogs [35] and are under development to treat human diseases. There is interest in creating viral vectors that encode factors inhibitory to HIV replication [36]. Unlike lentiviral vectors, which can be self-inactivating if they encode anti-HIV factors, FV vectors are not affected by anti-HIV proteins, making them a better choice for such approaches. Because FV are retroviruses, which undergo mutation and recombination, FV vectors should be carefully monitored for genetic changes that could be deleterious to hosts.

## Foamy virus natural infections

Foamy viruses have been isolated from many different animal hosts (Table 1). FV are ancient viruses that have coevolved with their natural hosts. The genomic sequence of an ancient relative of land animals, the coelacanth, was published in 2012 [37]. Remarkably, the genomic sequence revealed an endogenous foamy virus suggesting that foamy viruses have existed for at least 400 million years and that they are the oldest known eukaryotic viruses still extant. A comparison of the phylogenetic trees of foamy viruses and their primate hosts reveals congruence both in branching order and divergence time [8]. This indicates that FV have coevolved with nonhuman primates for at least 60 million years, essentially from the beginning of primate evolution.

**Table 1:** Foamy viruses and their natural hosts

Designation	Full name	Natural host	Original report
BFV	Bovine foamy virus	Cow	Malmquist et al. (1969) [41]
EFV	Equine foamy virus	Horse	Tobaly-Tapiero et al. (2000) [42]
FFV	Feline foamy virus	Domestic cat	Riggs et al. (1969) [43]
CFV	Chiropteran foamy virus	Bat	Wu et al. (2012) [38]
SFV	Simian foamy virus	Nonhuman primate (NHP)	Johnston et al. (1961) [44], Stiles et al. (1964) [45], Rogers et al. (1967) [46]
PFV (SFVpsc_huHSRV.13) [40]	Prototype foamy virus	Chimpanzee	Achong et al.(1971) [6]

As mentioned above, foamy viruses are mainly transmitted through saliva by biting, grooming and other means, such as sharing food. It is thus not surprising that the natural hosts for foamy viruses have life styles that include transfer of saliva between individuals. FV have been found in nonhuman primates and cats, which groom and bite members of their individual species. FV have also been described in cows, horses and at least one bat species (*Rhinolophus affinis*) [38]. Cows and horses share food sources with their herd members, for example, by chewing the same cud. Less is known about saliva transfer between bats. However, most bats are highly social and live in large groups, often sharing food. Bat social grooming has also been reported [39]. These behaviors likely lead to intra-species bat FV transmission. Bat FV is called CFV for chiropteran foamy virus [40].

Although there is no evidence for perinatal FV transmission, FV transmission from mothers to offspring occurs, most likely through breastmilk [4, 47]. In natural FV hosts such as cats, cows and NHP, juveniles do not appear to be productively infected and only become so as they mature [48–51]. A detailed study was done in Australia examining bovine foamy virus (BFV) transmission in herds of cattle [48]. The authors examined animals of different ages both for antibodies against BFV and for latent infection of PBMC. PBMC latent infection was defined by the ability of these cells to produce BFV after cocultivation with susceptible bovine cells (CLAB). Calves less than 6 months old, born to BFV positive mothers, were BFV antibody positive but CLAB negative while breastfeeding. Thus, the anti BFV antibodies in the calves were likely maternal. Early after weaning, when calves were separated from adults and pastured with other animals of similar ages and the same sex, anti BFV antibodies were no longer detected in the calves. However, by 18 months of age, the animals began producing BFV antibodies and became CLAB positive, indicative of SFV replication and viral spread to PBMC. A simple interpretation of these data is that newborn calves received both anti-BFV antibodies and virus from their mothers, but did not produce significant amounts of virus before weaning. After weaning and loss of maternal BFV antibodies, the calves began to produce their own anti-BFV antibodies caused by the onset of BFV replication and virus spread. Whether calves younger than 18 months old were unable to support BFV replication, or were protected from virus spread by maternal anti-BFV antibodies, remains unknown. From other studies, in adult macaques, it is known that PBMC containing latent SFV proviruses can transit to the oral mucosa, where susceptible superficial differentiating epithelial cells, once infected, can produce infectious SFV [9]. As discussed below, we favor the hypothesis that the calves were latently infected with maternal BFV but unable to support viral replication.

A similar picture emerged when a population of captive baboons was studied at a biomedical research foundation [52]. In this facility, infant baboons remained with their mothers for approximately 1 month, and then were removed to individual cages in a nursery without contact with other baboons. The infant baboons were initially SFV antibody positive but the antibody titers waned over the next 6 months. During the first 6 months, PBMC from the infant baboons were SFV PCR negative. PBMC from most juvenile baboons tested (ages not given, but presumably older than 6 months) were also SFV PCR negative. All adult baboons tested (5 years of age or older) were SFV antibody positive, and their PBMC were now SFV PCR positive, despite no interactions with other baboons. The only baboon-baboon interactions were with their mothers during their first month of life, which is when they became infected by SFV. As infants, the baboons acquired SFV maternal antibodies which should have prevented viral production and spread. Although the SFV antibodies waned, no juveniles showed signs of infection, which became evident only when the baboons reached adulthood. As in the cow study, a simple interpretation is that young baboons do not support productive SFV replication and spread. Longitudinal studies done in breeding facilities for *Macaca tonkeana* [53] and *Macaca fascicularis* [54] also showed that SFV infection increases with age and is not seen in animals younger than 6 months of age. Consistent with these results, studies in South and South East Asia showed that SFV antibody production increases with age in free-ranging macaques [51]. These authors had limited data on the SFV PCR status of the juvenile macaques but the data are consistent with PBMC SFV PCR-positivity increasing with age. A likely explanation for these findings is that over time more PBMC transit to the oral mucosa, the site of viral replication, where they become latently infected.

In summary, available data indicate that juvenile FV natural hosts are rarely, if ever, productively infected by FV. Only young adults and adult animals are productively infected. The extent of latent infection of PBMC, if it occurs at all, is at undetectable levels in juvenile animals. Either young juveniles lack host factor(s) required for FV replication or they have an inhibitory factor(s). This enigmatic point, that the time of initial exposure to FV does not coincide with ability to detect proviral DNA in PBMC by PCR or productive infection in the oral mucosa, must be considered when studying FV zoonotic infections.

Much less is known about SFV transmission in New World monkeys (NWM). An initial study, including three captive and four wild monkeys in Costa Rica, found that all seven NWM were SFV positive as detected by PCR [55]. The ages of these NWM were not reported. A study in Brasil [56] found SFV infections in 14 NWM genera including, howler monkeys (5 spp.), spider monkeys (2 spp.), owl monkeys (5 spp.), marmosets (10 spp.), capuchins (6 spp.), squirrel monkeys (3 spp.) and tamarins (9 spp.). Another Brazilian study [57], using both Western blot and PCR assays, found that SFV prevalence increases with age in different species of captive capuchin monkeys. Surprisingly, in wild NWM the SFV prevalence in sexually mature animals was somewhat lower than that in sexually immature animals (48 vs. 61%, respectively). These results indicate that sexual transmission of FV in NWM is unlikely.

In NHP, cross-species FV transmission occurs naturally. For example, in the Ivory Coast, SFV from Western red colobus monkeys was found in chimpanzees, which are predators of the colobus monkeys [27]. Similarly, chimpanzees are predators of *Cercopithecus* monkeys and a *Cercopithecus* SFV was detected in a wild chimpanzee in Equatorial Africa [28]. Phylogenetic analysis has revealed that cross-species and cross-genera transmissions of SFV also occur in NWM [56, 57, 26]. For example, several species of Cebus monkeys cohabit the same areas and SFV cross-species transmission was observed [26]. Such cross-species transmission may occur because of aggressive behaviors rather than predator–prey relationships. Cross-species transmission events in wild NHP could result in generation of new recombinant SFV strains. This is of concern since in the case of lentiviruses only mildly pathogenic SIV were found in African monkeys. After chimpanzees acquired several SIV strains through predation, recombination led to formation of SIVcpz which is somewhat pathogenic in chimpanzees and ultimately led to HIV in humans [29, 58].

FV infected gorillas and chimpanzees have been studied in Cameroon and Gabon [25]. Sequence analyses of the viral *env* genes revealed that there are at least two *env* strains in gorillas and chimpanzees. The data is consistent with recombination within species leading to viral strain differences, although not all the viral parental strains could be identified. Zoonotically infected humans living near chimpanzees and gorillas were found to be infected with the *env* recombinant viruses. However, there was no evidence for further viral recombination in the infected humans.

In addition to natural transmission of SFV in both free-living NHP and captive NHP in zoos and research laboratories, SFV can also be transferred by blood transfusion in research settings, as has been shown in *Macaca fascicularis* and *Macaca mulatta* [9, 59]. These studies in macaques have led to concerns that SFV could be transmitted to humans through blood transfusion from humans who have been in contact with NHP. In fact, it would be surprising if SFV were not transmitted via blood transfusion in humans, since latently infected PBMC would be present in the blood from an SFV infected donor.

## Viral coinfections in SFV natural hosts

Although there is no evidence that FV alone causes clinical disease, there is some indication that it may act as a cofactor in some other pathogenic infections. For example, several studies in macaques indicate that foamy viruses and lentiviruses interact in coinfected hosts. In one study, Murray et al. examined SFV replication in macaques that had been immunosuppressed as a consequence of infection with simian immunodeficiency virus (SIV) [60]. While in healthy macaques SFV replication was confined to the oral mucosa, in SIV immunosuppressed macaques SFV replication was also seen in the small intestine (jejunum). The jejunum is a major site of CD4+ T cell depletion upon SIV infection, but SFV replication was not seen in other tissues in which the number of CD4+ T cells was diminished. Choudhary et al. infected macaques with a laboratory created SIV strain (SIVmac239), highly pathogenic to macaques, and compared macaques naturally infected with SFV to uninfected animals [61]. The authors found that the SIVmac239 strain was more pathogenic in the SFV positive macaques. Specifically, in the SFV positive/SIV positive macaques the SIV viral load was higher, there were fewer CD4+ T cells, and a higher death rate was observed. Although the molecular basis for the SFV/SIV interaction is not known, this could have implications for HIV infected humans (see below).

## Foamy virus zoonotic infections

There are many situations in which humans come into contact with FV infected animals. Humans contact domestic cats, which in many countries are kept as pets. Most humans rarely contact cows, unless they live on a farm or work as a cattle farmer, but bovine products are widely consumed. Humans also contact horses in many situations. Interactions with NHP are frequent in research labs, breeding colonies and zoos, but can also occur with pet monkeys, which are often the smaller NWM species. In many parts of the world, such as Africa, Asia and South and Central America, humans often cohabit areas with NHP. Additionally, bush meat hunting is frequent in Africa. Thus, there are many interactions that could possibly lead to zoonotic transmission of feline foamy virus (FFV), bovine foamy virus (BFV), equine foamy virus (EFV) and simian foamy virus (SFV). A number of research groups have studied both the prevalence of antibodies to different FV species in humans as well as FV human persistent infections, as assayed by PCR.

### Non-primate hosts

In two studies, veterinarians who work with domestic cats were tested for FFV antibodies. In the first study 175 veterinarians were tested by ELISA and none were found to be FFV antibody positive [62]. A limitation of this study is that veterinarians were not interviewed about their exposure to cats through bites or scratches. In a second and more complete study, Butera et al. [63] examined 204 veterinarians for FFV antibodies using Western blots. Half of the veterinarians in this study reported > 17 years working with cats, and most of the veterinarians reported having cats as pets during their lifetime. In the year preceding the study, almost all participants had received cat bites, scratches or needle exposures to cat fluids. However, none of the subjects were FFV antibody positive. Therefore, there is currently no evidence for zoonotic transmission of FFV.

Since many humans consume bovine products, such as milk and beef, introduction of BFV into humans is also of interest. Unlike the case of FFV there are reports of BFV antibody positive humans. In one study veterinarians, dairy cow caretakers and cattle owners were screened for BFV antibodies. About 7% of the subjects were BFV antibody positive, indicating some exposure to the virus. However, none of these individuals were PCR positive for BFV DNA in PBMC [64]. In a more recent study, three groups of humans were screened for BFV antibodies. It was found that 7% of immunosuppressed patients, 38% of people who interact with cattle and 2% of the general population were BFV antibody positive. There was one BFV PCR positive subject in each group. Each short PCR product showed high homology to an US BFV isolate (M. Materniak-Kornas, personal communication). These data indicate that persistent BFV zoonotic infection is not common.

At present there is no published data showing CFV or EFV transmission to humans.

### Primate hosts

In contrast to the cases of FFV and BFV, there are many reports of zoonotic transmission of SFV. Interestingly, as discussed above, the original FV isolate, which was called HFV (human foamy virus), was isolated from a nasopharyngeal cancer cell line obtained from a Kenyan [6].The virus isolated from these cells was later determined to be of chimpanzee origin [7] and was renamed prototype foamy virus (PFV) [65]. PFV is also known as SFVpsc_ huHSRV.13 [40] but will be referred to as PFV herein. Since this original report, there have been many well-documented cases of SFV zoonotic infections, as detailed in Table 2.

**Table 2:** Representative SFV zoonotic infection studies

Location	Subjects	No.	No. SFV Ab+ (%)	No. SFV PCR+ (%)	SFV source	References
*A. North America*						
US and Canada	Lab workers exposed to NHP^a^	231	4 (1.8)	4 (1.8)	3 baboon 1 *Cercopithecus* sp.^c^	Heneine et al. [68]
North America	Zoo keepers working with NHP	133	4 (3)	N/A	Most likely chimpanzee	Sandstrom et al. [69]
Canada	Primate facility workers	46	2 (4.3)	1 (2.2)	1 macaque	Brooks et al. [70]
North America	Res. centers and zoo workers	187	10 (5.3)	9/9 Ab+ tested	8 chimpanzee 1 baboon	Switzer et al. [71]
*B. Africa*						
Cameroon	Bush meat hunters and butchers	1099	10 (0.9)	3 (0.3)	1 mandrill 1 gorilla 1 *Cercopithecus* sp.	Wolfe et al. [72]
Cameroon	VR^b^ near NHP populations	1164	21 (1.8)	4/11 Ab+ tested	3 gorilla 1 chimpanzee	Calattini et al. [73]
	Contact with NHP:					
	– apes	85 29	9 (10.6) 7 (24.1)	9 (10.6) 7 (24.1)	5 gorilla 2 chimpanzee	
	– monkeys	56	2 (3.6)	2 (3.6)	1 mandrill 1 *Cercopithecus* sp.	
Cameroon	General Adult population	1321	26 (2)	2 (0.2)	1 gorilla 1 *Cercopithecus* sp.	Betsem et al. [74]
	People with NHP bites or scratches	198	53 (26.7)	37 (18.6)	31 gorilla 3 chimpanzee 3 *Cercopithecus* sp.	
Gabon	NHP hunters and those interacting with pets^d^	78 10 women 59 men 9 children	19 (24.4)	15 (19.2)	12 gorilla 2 chimpanzee 1 *Cercopithecus* sp.	Mouinga-Ondéme et al. [75]
*C. Asia*						
Thailand, Indonesia, Nepal and Bangladesh	People sharing NHP habitat	305	8 (2.6)	3 (1)	3 macaques	Jones-Engel et al. [76]
Bangladesh	VR sharing NHP habitat	209	18 (8.6)	12 (5.6)	11 macaques	Engel et al. [77]

^a^Nonhuman primates, ^b^village residents

^c^Also known as guenons. This genus is comprised of at least 26 species of Old World monkeys

^d^Includes children

In many early studies, evidence for SFV zoonotic transmission was provided by the presence of human antibodies to SFV using Western blot assays. These early studies used SFV infected human cells to prepare lysates as targets for such antibodies. This led to many false positive results because the human antibodies often reacted to human rather than SFV proteins (reviewed in [50]). Later studies, using PCR assays [66] or more specific serological assays [51, 67] showed that SFV infection of humans was much less widespread than originally claimed. In particular, a study using SFV infected or uninfected monkey cells (Tf cells) eliminated most cross-reactivity issues in Western blot assays [51].

The presence of anti-Gag antibodies could be indicative of either persistent or transient SFV zoonotic infections. Persistent infection is defined by the presence of latent proviruses detected by PCR at least 1 year after initial infection. Transient infections produce anti-SFV antibodies, but do not have detectable levels of integrated provirus over time. In order to conclusively determine persistent infection it is customary to use PCR to detect the presence of integrated SFV provirus in peripheral blood mononuclear cells (PBMC) over time. Since the time of initial infection is not always precisely known, detection of proviral DNA at multiple time points can also indicate persistent infection.

Table 2 includes many of the published reports of SFV zoonoses from Old World monkeys (OWM). Three distinct groups of humans have been studied. The first group includes researchers and technicians who work with nonhuman primates (NHP) in Research Centers and Zoos in North America (Table 2A). The second group includes people in Africa who live in areas cohabited by NHP and also people who are NHP bushmeat hunters and/or butchers (Table 2B). Most of the people in these two groups are occupationally exposed to NHP and therefore could come in contact with NHP saliva and/or blood. The third group includes people in South Asia who cohabit areas with NHP and may or may not be occupationally exposed to NHP in temples (Table 2C).

#### Old World monkeys—North America

There have been at least four reports of SFV zoonotic infections of humans occupationally exposed to OWM and/or apes in laboratories and zoos in North America (Table 2A). In these studies the percent of SFV antibody positive people ranged from ca. 2–5% [68–71]. In three of these studies [68, 70, 71] the investigators examined the SFV PCR status of 16 SFV antibody positive humans and found that 14 were SFV PCR positive and one was SFV PCR negative. Blood was not available for DNA analysis from one subject. Thus, most of the SFV antibody positive subjects (14/15 or 93%) were persistently infected. Six spouses of these SFV PCR positive lab workers were tested and all were SFV antibody as well as SFV PCR negative [68, 71]. Boneva et al. examined the wives of six SFV positive North American men occupationally exposed to NHP and found that none of them were SFV antibody or PCR positive [78]. Thus, human to human transmission was not detected in North America.

#### Old World monkeys—Africa

As in North America, people in Africa are exposed to NHP at primate centers, including breeding colonies [79]. Unlike in North America, occupational exposure to NHP occurs in other situations such as hunting and butchering. In this regard, researchers have studied people in Cameroon and Gabon (Central Africa) who bushmeat hunt and people who prepare bushmeat for consumption; therefore, all these study subjects had direct contact with NHP or NHP tissues and/or fluids. In the case of butchers, it is possible that SFV transmission could occur through NHP blood rather than saliva. The researchers found that 11–27% of the individuals tested were SFV antibody positive, of which 72% were SFV PCR positive [72–75] (Table 2B). In contrast, when researchers studied Africans who cohabit areas with NHP, less than 0.5% of people screened were SFV antibody positive [73, 74] (Table 2B). Of these antibody positive people, 16% were persistently infected as detected by PCR. There were also studies done in Western African countries, Democratic Republic of Congo and Ivory Coast. In Ivory Coast, the researchers studied a large number of people some of whom were HIV infected and found that 3 of the people (0.2%) were SFV antibody positive. 2 of the SFV positive people were HIV positive, demonstrating HIV and SFV co-infection [80]. In the Democratic Republic of Congo over 3000 villagers were screened for SFV infection and 0.5% were found to be SFV antibody positive using Western blots [81]. Compared to the laboratory workers in North America, who presumably take universal precautions, a far greater percent of Africans in direct contact with NHP, primarily hunters, showed exposure to SFV, as measured by antibody production.

In one study the participants were classified by whether they had direct contact with apes or with monkeys [73]. Those who encountered apes were about six times more likely to be SFV infected than those who encountered monkeys. Although the specifics of these encounters are not known, it is likely that ape SFV is more easily zoonotically transmitted than monkey SFV. Since ape bites are deeper than monkey bites, this could result in more efficient transfer of virus in saliva to humans. Consistent with this idea, in two studies where PCR results were obtained, most SFV PCR positive people in Africa were persistently infected with a gorilla SFV [74, 75]. Whether transmission through blood could play a role in more efficient viral transfer from ape to humans has not been examined.

Calattini et al. [73] looked at five spouses and five children of SFV PCR positive subjects in Africa and found that none of the relatives were infected. Betsem et al. [74] examined 30 spouses of SFV PCR positive African subjects and one of the spouses was SFV antibody positive but SFV PCR negative. Because this woman was PCR negative, it was not possible to determine whether the SFV antigen source was her husband or an NHP she independently encountered. Switzer et al. also look at relatives (spouses, parents, siblings and offspring) of 8 SFV antibody positive women in the Democratic Republic of Congo and found no evidence of SFV infection [81]. Overall, no evidence for intra-familial transmission of SFV was found in Africa.

#### Old World monkeys—Asia

SFV zoonotic transmission has also been studied in Asia (Table 2C). In Asia, there are apes such as gibbons and orangutans but humans who interact with these apes have not been studied. In the areas studied (Thailand, Indonesia, Nepal and Bangladesh) the primary NHP genus is *Macaca* (macaques). In one study people who cohabit areas with macaques in these four countries were analyzed [76]. It was found that 2.6% of the people screened were SFV antibody positive and 1% were SFV PCR positive. Most of the people screened were exposed to macaques in temples. Work then focused on people in Bangladesh, one of the world’s most densely populated countries, where a large percentage of the population cohabits areas with macaques. Unlike in Africa, people in Bangladesh do not hunt or consume NHP. In the Bangladesh study, about half of 209 village residents (VR) who were sampled reported having been bitten at least once by a macaque. 8.6% of the 209 VR were SFV antibody positive and 5.6% SFV PCR positive.

In orthoretroviruses, such as murine leukemia virus (MLV), the *env* gene appears to be the most diverse gene [82]. MLV are classified by their host range, which is defined by the receptor binding domain of the Env protein. In contrast, in foamy viruses the *gag* gene sequence is very variable and can be used to define viral strains [24]. The SFV proviruses in Bangladesh macaques were sequenced and six SFV strains were defined based on sequence variation in the *gag* gene. 25% of the adult macaques sampled harbored at least two distinct SFV strains. Humans were found who were also infected with more than one SFV strain. Three humans were coinfected with strain combinations not seen in individual macaques, suggesting that the SFV strains detected were zoonotically transmitted from more than one macaque. This could lead to generation of new recombinant strains in humans, as has been reported to occur in macaques [24]. Such recombination could occur since there is evidence that some SFV replication does occur in humans (see below for discussion). Some of the VR were sampled at two time points (about 1 year apart) and the SFV proviruses were sequenced. In some of these VR, for whom more than one SFV strain was detected by PCR in the initial time point, there were differences in the abundance of strains recovered at the second time [77]. These participants did not report any interactions with NHP between the two time points. Therefore, it is unlikely that they became infected with a different SFV strain between the two sampling times. It is possible that acquired or innate immunity factors could influence the persistence of different SFV strains in humans over time. This requires further study. The difference in abundance of SFV strains over time implies that viral replication does occur in humans. Other evidence for SFV replication in humans is discussed below.

Very little is known about the age at which humans are susceptible to SFV zoonotic infection. The studies in Asia only included adults ages18 or older. One SFV positive 19 year-old Bangladeshi woman reported being severely bitten by a macaque when she was 4 years old. However, as a young adult, macaques did enter her house leaving behind urine and feces. It is likely that she was infected as a child but this has not been validated [76]. Further studies need to be done to determine at what age humans can become persistently infected by SFV. It is also of interest to determine whether there is any age restriction on viral replication as appears to be the case in cows and NHP.

In most of the studies reported in Table 2 the subjects were tested for antibodies to SFV using Western blot or ELISA, and for persistent infection using PCR of DNA extracted from PBMC. There are humans who have been exposed to SFV and who are SFV antibody positive but are not persistently infected, as detected by an SFV PCR assay. People who are SFV PCR positive are of interest because they have the potential to transmit virus to other humans via saliva. It is known that SFV replication occurs in the oral mucosal epithelia cells of NHP [32]. Viral replication would presumably have to occur in the human oral mucosa for efficient FV human to human transmission. When foamy viruses replicate a large amount of viral RNA is produced and can be detected by reverse transcriptase-PCR (RT-PCR). Engel et al. used a sensitive and quantitative RT-PCR assay to look for SFV RNA in buccal swabs from Bangladeshi humans who were persistently infected by macaque SFV, as detected by PCR [77]. Although SFV RNA could easily be detected in macaque buccal swabs using the same assay [83] no SFV RNA could be detected in the human buccal swabs. Similarly, no RNA was detected in buccal swabs from Africans infected by gorilla SFV [84]. These data indicate that SFV replication does not occur in human oral mucosa to the same extent as in NHP oral mucosal tissue. Thus, SFV transfer between humans via saliva is unlikely. PBMC from SFV PCR positive Bangladeshi humans were also assayed for SFV RNA and none was detected [83]. Boneva et al. [78] looked for infectious virus in saliva samples from six SFV positive people in North America. They cultured virus from the saliva of one individual but only in one of four attempts. In this individual PCR analysis of DNA clones from different body sites indicated SFVcpz quasispecies [85]. Thus, there is indirect evidence for SFV replication in this human.

Although there is no direct evidence for SFV replication in humans, as measured by SFV RNA synthesis, several findings can only be explained by some level of SFV viral replication. As cited above, SFV quasispecies was found in a human, with viral DNA sequences varying between tissue compartments [85]. Matsen et al. sequenced proviruses from PBMC obtained from SFV PCR positive humans [86]. Interestingly, evidence for APOBEC3 deamination was found in some proviruses. When SFV proviruses were analyzed in the macaques with which the humans interacted, no evidence of APOBEC3 deamination could be found. Because APOBEC3 deamination occurs during SFV replication, at the single strand DNA synthesis step of reverse transcription, these results suggest that some SFV replication occurs in humans. SFV replication in humans can also be inferred from the large number of human PBMC positive for FV proviral DNA by PCR. Given that about one SFV proviral DNA copy is found per 10^4^ PBMC [55] and that the average human has ca. 5 × 10^9^ PBMC, this would require the transfer of about 10^5^ infectious SFV particles. This high level of virus is unlikely to be transferred during an NHP bite or scratch. Finally, when samples were taken from SFV-infected humans at different times, the dominant strains differed in the same individual [77]. While this could arise from secondary SFV infection at a time distal from initial infection, there is no evidence for this in the individuals cited. This implies that some strains replicate or persist better than others in different individuals.

In Bangladesh and other South Asian countries, there are ethnically homogenous seminomadic human groups, known as the Bedey. There is a group of Bedey residing in Northeastern Bangladesh who interact more with macaques than do the villagers residing in the same area. This particular Bedey group in Bangladesh trains macaques for performances. These Bedey also earn their livelihoods by performing with these animals. Despite constant interactions with macaques, often leading to bites and scars, these Bedey do not appear to be susceptible to SFV infection. A small number (*n* = 45) of the Northeast Bangladesh Bedey were screened and none of them were SFV antibody positive or SFV PCR positive [87]. These findings suggest that these Bedey constitute a unique human group with high exposure to NHP who may be resistant to SFV infection. The prevalence of SFV in the Bedey performing macaques is nearly as high as that seen in the free-ranging population of macaques in Bangladesh [51]. The SFV viral strains in the performing and free-ranging macaque groups are very similar, based on *gag* sequences [24]. The difference in zoonotic transmission of SFV between Bedey and village residents (VR) who live in the same areas seems compelling. However, as only 45 Bedey were tested, the observed difference in SFV prevalence is not statistically significant (*p* value < 0.078). Macaque bites often lead to scars and the number of scars in the Bedey and VR were estimated by visual inspection and interviews. There were 152 scars in 269 VR screened, whereas the 45 Bedey had a total of 297 scars (Jones-Engel et al., unpublished results). When the number of SFV PCR positive humans relative to the total number of scars was compared, 12 SFV PCR positive humans per 152 total scars were found in the VR group vs 0 SFV PCR positive humans per 297 total scars in the Bedey group. This difference is statistically significant (*p* value < 0.0001).

In any human group interacting with OWM, only a fraction of the humans are SFV infected as measured by PCR. Thus, if it can be determined why the Bedey are resistant to SFV infection these results could be extended to other human groups. It is not known why the Bedey are both SFV antibody and SFV PCR negative, but it could be a result of either acquired or innate immunity. For example, it is known that APOBEC3G can modify SFV genomes in humans [86]. Perhaps the Bedey have very active APOBEC3 genes, which could lead to inactivation of SFV proviruses. Inactivation of proviruses will ultimately shut-down any low level of replication suspected to occur in other humans (innate immunity). Another possibility is that the Bedey have high levels of anti-SFV neutralizing antibodies (acquired immunity). Currently, Western blots only detect anti-SFV Gag antibodies, but not anti-SFV Env antibodies. High levels of anti-SFV Env antibodies could neutralize virus and prevent infection. Until more Bedey are screened, acquired or innate immunity differences between Bedey and VR are merely speculative.

#### New World monkeys

Table 2 does not include any examples of zoonotic infection by New World monkey (NWM) SFV. In fact, there are currently no reports of such infections. NWM SFV can infect and replicate in human-derived tissue culture cells [88, 55], demonstrating that there is no intrinsic block to replication of NWM SFV in human cells. To date, only a few studies have been published in which NWM SFV zoonotic transmission was examined. In the first published study, a group of primatologists known to have been exposed to NWM was screened by both Western blot and PCR [55]. The nested *pol* PCR assay used could detect SFV DNA in the blood of squirrel, howler and capuchin monkeys as well as from TC cells infected with spider monkey and marmoset SFV. The Western blot antigen used in this study, spider monkey SFV proteins, could be detected using plasma from squirrel monkeys. This antigen was also used to determine whether anti-NWM SFV antibodies could be detected in humans. A total of 69 primatologists were examined and 11.6% (8/69) had antibodies reactive to spider monkey SFV. While all of the 8 NWM SFV seropositive individuals reported some contact with NWM species, only 4 reported direct contacts, such as bites, scratches, or needle sticks. The remaining 4 individuals reported indirect contacts with NWM, including exposure to body fluids. Unlike most OWM SFV-seropositive individuals, NWM SFV-seropositive individuals did not have detectable levels of viral DNA in their blood as assayed by the highly sensitive nested PCR assay which can detect SFV DNA from at least 5 NWM genera. The NWM species that the primatologists interacted with in this study are not known, however the Western blot assay used detects at least spider and squirrel monkey antibodies. Moreover, the PCR assay detects at least, spider, squirrel, capuchin, marmoset and howler monkey SFV DNA. Thus, the PCR assay can detect more NHP genera than the Western blot assay and the humans who tested NWM SFV Ab positive were SFV PCR negative.

In another published study, 56 people occupationally exposed to NWM in Brazil were screened for NWM SFV infection using both antibody (Western blot) and PCR assays [89]. In this longitudinal study 18% (10/56) of the people sampled were NWM SFV antibody positive at the initial time point, but none of these seropositive people were SFV PCR positive. Six of the 10 seropositive individuals remained seroreactive when screened 2–3 years later. Interestingly three individuals seroreverted, for unknown reasons. In summary, human exposure to NWM SFV occurs and leads to anti-SFV antibody production, however to date there is no evidence of persistent infection of humans with NWM SFV, as detected by PCR.

## Viral coinfections in SFV infected humans

Two independent studies in Africa reported a total of four individuals coinfected with SFV and HIV in Cameroon and the Ivory Coast [90, 80]. PBMC DNA was available from two individuals. One was infected with a mandrill SFV and the other one with a guenon SFV. However, there was no mention of the disease outcomes of these coinfected humans relative to humans infected with only HIV.

As discussed above it is known that in macaques there are interactions between foamy virus and lentiviruses that exacerbate the pathogenicity of lentiviruses. Thus, the possibility arises that SFV infection in humans could also augment HIV pathogenicity. Nothing is known about other viral infections, such as CMV, in people who are infected with SFV, but this warrants further study.

## Conclusions, speculation and perspectives

Foamy viruses are ancient retroviruses that have apparently existed for at least 400 million years. They predate all other known retroviruses and hepadnaviruses, their closest relatives. Unlike orthoretroviruses, but similar to hepadnaviruses, infectious foamy viruses have functional DNA genomes.

There is much interest in developing SFV as gene therapy vectors for treatment of human diseases. Foamy viruses are retroviruses that permanently integrate into cell genomes and can be used for long-lasting expression of genes of interest. As the functional FV genome is DNA, FV vectors are more likely to be more chemically stable than vectors with RNA genomes, such as orthoretroviruses. These features, combined with a lack of pathogenicity, make SFV promising candidates for development of viral vectors. Foamy viruses are highly prevalent in their natural hosts, which include bats, cats, cows, horses and NHP. Studies in cows and NHP indicate that foamy virus does not replicate efficiently in juveniles, but latent viral infection seems likely and the viral source is probably of maternal origin, perhaps through breast milk. In the same host species, productive FV infection is detected in almost all adults. Why there is a difference in FV replication between juveniles and adults is not known, but this is an area that should be explored further, especially as it has implications for the development of FV gene therapy vectors for humans.

Zoonotic transmission of SFV is not very widespread because most humans do not interact directly with NHP. However, in those humans who interact directly with Old World monkeys and apes, SFV is rather easily zoonotically transmitted and infected humans do not appear to have any pathology associated with SFV infection. Humans who are SFV Gag antibody positive can be either SFV PCR positive or SFV PCR negative when PBMC DNA is assayed. This indicates that humans who encounter SFV and produce SFV Gag antibodies are not always persistently infected but instead could be transiently infected. A group of humans, the Bedey of Northeastern Bangladesh, frequently interact with macaques and often have scars from macaque bites. Despite these interactions, these Bedey do not appear to be either SFV Gag antibody positive or SFV PCR positive. Understanding human-encoded factors important for establishing SFV persistent infection, which does not seem to occur in these Bedey, is important for the development of optimal gene therapy vectors and may offer new insights into anti-retroviral strategies.

The source of SFV (apes, Old World monkeys or New World monkeys) is an important factor in SFV zoonotic transmission. Humans appear not to be persistently infected by NWM SFV and more susceptible to ape SFV than to OWM SFV infection. This suggests that humans are most sensitive to SFV strains from NHP to which they are more genetically related. It is interesting to speculate as to why humans do not have their own FV, although chimpanzees, the closest NHP relative to humans, do. A likely explanation is that saliva transfer into the blood stream is rarer among adult humans than among adult chimpanzees and other NHP. NHP grooming behaviors, unlike those of humans, routinely involve scratching and biting with saliva transfer. Also, using their opposable thumbs, humans have developed tools that replace biting as an offensive or defensive adult behavior. The paucity of saliva transmission among adult humans might explain the lack of an endemic human FV.

Active foamy virus replication is detected by measuring viral RNA production. To date no viral RNA in either oral mucosal tissue or blood cells has been detected in SFV-infected humans. However, several lines of evidence suggest that some level of SFV replication must occur in humans. Understanding the timing and location of this undetectable SFV replication in humans will be helpful in monitoring the impact and risk of zoonotic infections.

Although SFV is not known to be pathogenic in humans, nothing has been done to determine whether SFV infections in humans can exacerbate other viral infections. In macaques, SFV infection can accelerate disease by pathogens such as lentiviruses. Macaques infected with a modified SIV in research settings are sicker and die sooner if they are also naturally SFV infected. It is possible that humans who are SFV positive are also prone to accelerated disease by human pathogenic viruses but this has not been studied. Given the number of people interacting with NHP around the world, this is an area of concern.

As some humans are coinfected with more than one SFV strain, they should be closely monitored for the appearance of recombinant SFV strains, as well as for any pathological consequences of infection. Lentiviruses are not highly pathogenic in their natural hosts, but recombination has been shown to generate strains that are pathogenic in accidental hosts such as humans. Understanding the determinants of SFV zoonotic transmission is critical as there are concerns that SFV could emerge as a new human pathogen.

All viruses require transmission in order to survive, and in this respect, foamy viruses could be considered “perfect” viruses. In natural populations foamy viruses spread efficiently to reach very high prevalence rates. Many viruses induce symptoms deleterious to the host in order to be efficiently transmitted. For example, nasopharyngeal viruses often induce runny noses, sneezing and coughing to aid in their transmission. Foamy viruses are mainly transmitted through saliva, and all natural hosts have saliva transfer as part of their regular life style. Thus, foamy viruses do not need to induce any pathological symptoms to aid in their transmission.

In summary, foamy viruses are the most ancient retroviruses and have a long history of coevolution with their natural hosts. Viral transmission occurs efficiently within natural populations to establish life-long, non-pathogenic infections. Zoonotic transmission of SFV can also lead to persistent infection in humans, although less frequently than is seen in natural hosts. Given their unique genomic features and lack of pathogenicity in humans, SFV continue to show promise as vectors for the treatment of life-threatening diseases.
